# (2*E*)-*N*-Methyl-2-[(2*E*)-3-phenyl­prop-2-en-1-yl­idene]hydrazinecarbothio­amide

**DOI:** 10.1107/S1600536812037397

**Published:** 2012-09-05

**Authors:** P. Murali Krishna, G. N. Anilkumar, K. Hussain Reddy, M. K. Kokila

**Affiliations:** a Department of Chemistry, M. S. Ramaiah Institute of Technology, M.S.R.I.T. Post, Bangalore 560 054, Karnataka, India; b Department of Physics, M. S. Ramaiah Institute of Technology, M.S.R.I.T. Post, Bangalore 560 054, Karnataka, India; c Department of Chemistry, Sri Krishnadevaraya University, Anantapur 515 003 (AP), India; d Department of Physics, Bangalore University, Bangalore 560 056, Karnataka, India

## Abstract

The title compound, C_11_H_13_N_3_S, is close to being planar, with a dihedral angle of 9.64 (3)° between the benzene ring and the thio­semicarbazone mean plane, maintained by the presence of π-conjugation in the chain linking the the two systems. In the crystal, N—H⋯S hydrogen bonds form centrosymmetric dimers through a cyclic association [graph-set *R*_2_^2^(8)].

## Related literature

For the biological activity and pharmaceutical properties of thio­semicarbazones and their derivatives, see: Casas *et al.* (2000 ▶); Ferrari *et al.* (2000 ▶); Murali Krishna *et al.* (2008 ▶); Murali Krishna & Hussain Reddy (2009 ▶). For bond-length data, see: Allen *et al.* (1987 ▶). For hydrogen-bond motifs, see: Bernstein *et al.* (1995 ▶). For related compounds, see: Chumakov *et al.* (2006 ▶).
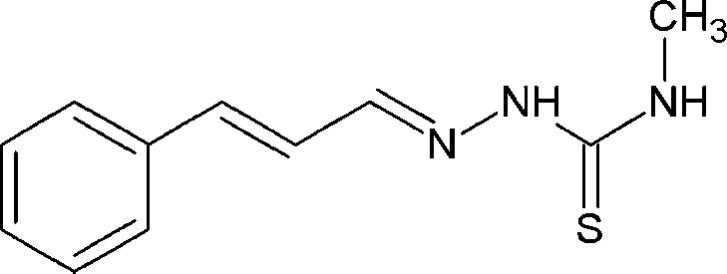


## Experimental

### 

#### Crystal data

C_11_H_13_N_3_S
*M* *_r_* = 219.31
Monoclinic, 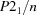

*a* = 5.5265 (11) Å
*b* = 9.4670 (19) Å
*c* = 22.534 (5) Å
β = 91.206 (3)°
*V* = 1178.7 (4) Å^3^
*Z* = 4
Mo *K*α radiation
μ = 0.25 mm^−1^
*T* = 291 K
0.45 × 0.26 × 0.24 mm

#### Data collection

Bruker SMART CCD area-detector diffractometer
8363 measured reflections
2201 independent reflections
1564 reflections with *I* > 2σ(*I*)
*R*_int_ = 0.031

#### Refinement

*R*[*F* ^2^ > 2σ(*F* ^2^)] = 0.059
*wR*(*F* ^2^) = 0.174
*S* = 1.06
2201 reflections
137 parameters
H-atom parameters constrained
Δρ_max_ = 0.38 e Å^−3^
Δρ_min_ = −0.32 e Å^−3^

### 

Data collection: *SMART* (Bruker, 2007 ▶); cell refinement: *SAINT* (Bruker, 2007 ▶); data reduction: *SAINT*; program(s) used to solve structure: *SIR92* (Altomare *et al.*, 1993 ▶); program(s) used to refine structure: *SHELXL97* (Sheldrick, 2008 ▶); molecular graphics: *ORTEP-3* (Farrugia, 1997 ▶) and *CAMERON* (Watkin *et al.*, 1993 ▶); software used to prepare material for publication: *PARST* (Nardelli, 1995 ▶) and *WinGX* (Farrugia, 1999 ▶).

## Supplementary Material

Crystal structure: contains datablock(s) I, global. DOI: 10.1107/S1600536812037397/zs2229sup1.cif

Structure factors: contains datablock(s) I. DOI: 10.1107/S1600536812037397/zs2229Isup2.hkl

Supplementary material file. DOI: 10.1107/S1600536812037397/zs2229Isup3.cml

Additional supplementary materials:  crystallographic information; 3D view; checkCIF report

## Figures and Tables

**Table 1:** Hydrogen-bond geometry (Å, °)

*D*—H⋯*A*	*D*—H	H⋯*A*	*D*⋯*A*	*D*—H⋯*A*
N2—H2⋯S1^i^	0.86	2.67	3.368 (3)	139

Symmetry code: (i) 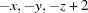.

**Fig. 1.**
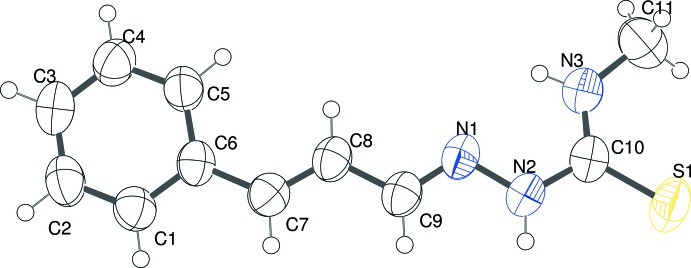
The structure of the title compound showing atom numbering, with probability ellipsoids drawn at the 50% level.

**Fig. 2.**
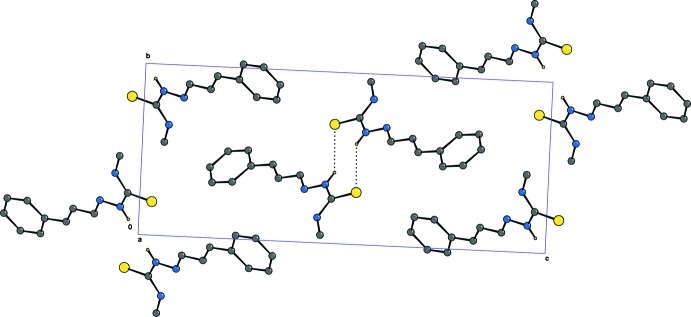
The packing diagram showing the molecular dimers with N— H···S interactions shown as dashed lines. H-atoms not involved in H-bonding are omitted.

## supplementary crystallographic information

### Comment

Thiosemicarbazones, the derivatives of carbonyl compounds with thiosemicarbazides have been given a special place among sulfur containing compounds due to their propensity to react with a wide range of metals (Casas *et al.*, 2000) and possess a wide spectrum of medicinal properties (Ferrari *et al.*, 2000). It was advocated that their bioactivity arises because of the presence of the imino group (–N═CH–) in addition to thioamino moieties present in the skeleton of the molecule. The title thiosemicarbazone derivative, C_11_H_13_N_3_S was synthesized and its crystal structure is reported here. It is possible that this compound may have biomedical properties similar to other nitrogen-sulfur donor ligands studied by our group.

In the title compound (Fig. 1), the aromatic ring and the thiosemicarbazone moiety are *anti*-related about the C7═C8 bond and the molecule is almost planar, with maximum deviations from the l.s. plane of -0.127 (3) Å (N2) and 0.135 (5) Å (N3). The dihedral angle between the benzene ring and the thiosemicarbazone plane is 9.64 (2)°. The result is the presence of π-conjugation between the aromatic system and the thiosemicarbazide fragment of the molecule. All bond lengths and angles are normal (Allen *et al.*, 1987). A comparison of the interatomic distances of C10—S1, C2—N2 and N2—N1 and the variations in bond angles N2–C10–N3 [115.8 (2)°], N2—C10—S1 [119.7 (2)°] and N3–C10–S1 [124.5 (2)°] in the ligand, indicate that in the TSC moiety of this substituted thiosemicarbazone there is extensive electron lone pair delocalization. The molecular conformation of this group is stabilized by intramolecular N3—H···N1 and C11—H···S1 interactions giving *S*(5) motifs (Bernstein *et al.*, 1995). Intermolecular N—H···S hydrogen bonds (Table 1) give centrosymmetric dimers through a cyclic association [graph set *R*^2^_2_(8)] (Fig. 2).

### Experimental

To a hot ethanolic (25 ml) solution of (2*E*)-3-phenylprop-2-enal (4.9 g, 0.03 mol) was added 50 ml of *N*-methylhydrazinecarbothioamide (0.03 mol) in 5% aqueous acetic acid (50 ml) and the reaction mixture was refluxed for 30–45 min. The crystalline solid product formed was collected by filtration, washed 5–6 times with 10 ml of hot water and then dried under vacuum and re-crystallized from methanol (20 ml). Good diffraction-quality pale yellow crystals of the title compound were obtained in a 1:1 mixture of ethanol and *n*-hexane.

### Refinement

All H atoms were fixed geometrically and treated as riding with N—H = 0.86 Å, C—H = 0.93 Å (aromatic) or 0.96 Å (methyl) with *U*_iso_(H) = 1.2*U*_eq_(N, C_aromatic_) or *U*_iso_(H) = 1.5*U*_eq_(C_methyl_).

### Crystal data

**Table:**

C_11_H_13_N_3_S	*F*(000) = 464
*M**_r_* = 219.31	*D*_x_ = 1.236 Mg m^−^^3^
Monoclinic, *P*2_1_/*n*	Mo *K*α radiation, λ = 0.71073 Å
Hall symbol: -P 2yn	Cell parameters from 290 reflections
*a* = 5.5265 (11) Å	θ = 1.5–26.8°
*b* = 9.4670 (19) Å	µ = 0.25 mm^−^^1^
*c* = 22.534 (5) Å	*T* = 291 K
β = 91.206 (3)°	Needle, pale yellow
*V* = 1178.7 (4) Å^3^	0.45 × 0.26 × 0.24 mm
*Z* = 4	

### Data collection

**Table:**

Bruker SMART CCD area-detector diffractometer	1564 reflections with *I* > 2σ(*I*)
Radiation source: fine-focus sealed tube	*R*_int_ = 0.031
Graphite monochromator	θ_max_ = 25.5°, θ_min_ = 1.8°
ψ and ω scans	*h* = −6→6
8363 measured reflections	*k* = −11→11
2201 independent reflections	*l* = −27→27

### Refinement

**Table:**

Refinement on *F*^2^	Primary atom site location: structure-invariant direct methods
Least-squares matrix: full	Secondary atom site location: difference Fourier map
*R*[*F*^2^ > 2σ(*F*^2^)] = 0.059	Hydrogen site location: inferred from neighbouring sites
*wR*(*F*^2^) = 0.174	H-atom parameters constrained
*S* = 1.06	*w* = 1/[σ^2^(*F*_o_^2^) + (0.0883*P*)^2^ + 0.2979*P*] where *P* = (*F*_o_^2^ + 2*F*_c_^2^)/3
2201 reflections	(Δ/σ)_max_ = 0.001
137 parameters	Δρ_max_ = 0.38 e Å^−^^3^
0 restraints	Δρ_min_ = −0.32 e Å^−^^3^

### Special details

**Table:**

Geometry. All e.s.d.'s (except the e.s.d. in the dihedral angle between two l.s. planes) are estimated using the full covariance matrix. The cell e.s.d.'s are taken into account individually in the estimation of e.s.d.'s in distances, angles and torsion angles; correlations between e.s.d.'s in cell parameters are only used when they are defined by crystal symmetry. An approximate (isotropic) treatment of cell e.s.d.'s is used for estimating e.s.d.'s involving l.s. planes.
Refinement. Refinement of *F*^2^ against ALL reflections. The weighted *R*-factor *wR* and goodness of fit *S* are based on *F*^2^, conventional *R*-factors *R* are based on *F*, with *F* set to zero for negative *F*^2^. The threshold expression of *F*^2^ > σ(*F*^2^) is used only for calculating *R*-factors(gt) *etc*. and is not relevant to the choice of reflections for refinement. *R*-factors based on *F*^2^ are statistically about twice as large as those based on *F*, and *R*- factors based on ALL data will be even larger.

### Fractional atomic coordinates and isotropic or equivalent isotropic displacement parameters (Å^2^)

**Table:**

	*x*	*y*	*z*	*U*_iso_*/*U*_eq_	
S1	−0.18251 (17)	0.19579 (9)	1.02989 (4)	0.0887 (4)	
N1	0.2855 (4)	0.1898 (2)	0.90259 (9)	0.0638 (6)	
N2	0.1638 (4)	0.1576 (3)	0.95336 (10)	0.0675 (6)	
H2	0.2067	0.086	0.9746	0.081*	
N3	−0.0653 (4)	0.3521 (3)	0.93696 (10)	0.0709 (7)	
H3	0.0296	0.3671	0.9079	0.085*	
C1	1.1293 (5)	−0.0205 (3)	0.76071 (12)	0.0668 (7)	
H1	1.1667	−0.0888	0.7891	0.08*	
C2	1.2696 (5)	−0.0095 (4)	0.71108 (13)	0.0764 (8)	
H2A	1.4009	−0.0698	0.7066	0.092*	
C3	1.2180 (5)	0.0884 (3)	0.66856 (14)	0.0753 (8)	
H3A	1.3135	0.0957	0.6352	0.09*	
C4	1.0231 (6)	0.1766 (3)	0.67547 (14)	0.0760 (8)	
H4	0.9867	0.2435	0.6464	0.091*	
C5	0.8811 (5)	0.1673 (3)	0.72468 (13)	0.0685 (7)	
H5	0.7493	0.2275	0.7283	0.082*	
C6	0.9323 (4)	0.0688 (3)	0.76925 (11)	0.0571 (6)	
C7	0.7899 (4)	0.0553 (3)	0.82258 (11)	0.0616 (7)	
H7	0.8363	−0.0157	0.849	0.074*	
C8	0.6004 (5)	0.1334 (3)	0.83780 (12)	0.0626 (7)	
H8	0.5532	0.2071	0.8128	0.075*	
C9	0.4655 (5)	0.1101 (3)	0.89050 (11)	0.0627 (7)	
H9	0.5088	0.0368	0.9161	0.075*	
C10	−0.0242 (5)	0.2395 (3)	0.96975 (12)	0.0621 (7)	
C11	−0.2592 (6)	0.4535 (4)	0.94608 (16)	0.0907 (10)	
H11A	−0.4034	0.422	0.9256	0.136*	
H11B	−0.2123	0.544	0.9309	0.136*	
H11C	−0.2896	0.4613	0.9877	0.136*	

### Atomic displacement parameters (Å^2^)

**Table:**

	*U*^11^	*U*^22^	*U*^33^	*U*^12^	*U*^13^	*U*^23^
S1	0.1036 (7)	0.0873 (6)	0.0769 (6)	−0.0033 (5)	0.0421 (5)	−0.0020 (4)
N1	0.0603 (13)	0.0759 (15)	0.0558 (13)	−0.0073 (11)	0.0119 (10)	−0.0001 (11)
N2	0.0673 (14)	0.0775 (15)	0.0582 (13)	0.0019 (12)	0.0148 (11)	0.0093 (11)
N3	0.0657 (14)	0.0768 (15)	0.0705 (14)	−0.0019 (12)	0.0103 (11)	0.0037 (12)
C1	0.0565 (15)	0.0770 (18)	0.0668 (17)	0.0015 (13)	−0.0017 (13)	−0.0026 (14)
C2	0.0565 (16)	0.095 (2)	0.077 (2)	0.0084 (15)	0.0084 (14)	−0.0134 (17)
C3	0.0700 (18)	0.085 (2)	0.0713 (18)	−0.0077 (16)	0.0201 (14)	−0.0150 (16)
C4	0.084 (2)	0.0718 (19)	0.0728 (19)	−0.0015 (16)	0.0184 (15)	0.0046 (14)
C5	0.0659 (16)	0.0680 (17)	0.0722 (18)	0.0046 (13)	0.0148 (13)	0.0011 (14)
C6	0.0461 (13)	0.0651 (15)	0.0601 (15)	−0.0095 (12)	0.0042 (11)	−0.0082 (12)
C7	0.0517 (14)	0.0700 (16)	0.0628 (15)	−0.0083 (12)	−0.0008 (12)	0.0007 (13)
C8	0.0555 (15)	0.0743 (17)	0.0581 (15)	−0.0084 (13)	0.0056 (12)	−0.0023 (13)
C9	0.0577 (15)	0.0745 (18)	0.0560 (15)	−0.0083 (14)	0.0034 (12)	−0.0030 (13)
C10	0.0608 (15)	0.0658 (16)	0.0601 (16)	−0.0071 (13)	0.0094 (12)	−0.0074 (13)
C11	0.076 (2)	0.081 (2)	0.115 (3)	0.0053 (16)	0.0029 (18)	0.0001 (19)

### Geometric parameters (Å, º)

**Table:**

S1—C10	1.680 (3)	C3—H3A	0.93
N1—C9	1.282 (3)	C4—C5	1.375 (4)
N1—N2	1.373 (3)	C4—H4	0.93
N2—C10	1.354 (3)	C5—C6	1.395 (4)
N2—H2	0.86	C5—H5	0.93
N3—C10	1.314 (4)	C6—C7	1.456 (3)
N3—C11	1.456 (4)	C7—C8	1.333 (4)
N3—H3	0.86	C7—H7	0.93
C1—C2	1.378 (4)	C8—C9	1.432 (4)
C1—C6	1.394 (4)	C8—H8	0.93
C1—H1	0.93	C9—H9	0.93
C2—C3	1.359 (4)	C11—H11A	0.96
C2—H2A	0.93	C11—H11B	0.96
C3—C4	1.374 (4)	C11—H11C	0.96
			
C9—N1—N2	116.3 (2)	C1—C6—C5	116.9 (2)
C10—N2—N1	119.5 (2)	C1—C6—C7	119.8 (2)
C10—N2—H2	120.2	C5—C6—C7	123.2 (2)
N1—N2—H2	120.2	C8—C7—C6	127.2 (3)
C10—N3—C11	125.0 (3)	C8—C7—H7	116.4
C10—N3—H3	117.5	C6—C7—H7	116.4
C11—N3—H3	117.5	C7—C8—C9	123.6 (3)
C2—C1—C6	121.4 (3)	C7—C8—H8	118.2
C2—C1—H1	119.3	C9—C8—H8	118.2
C6—C1—H1	119.3	N1—C9—C8	120.3 (3)
C3—C2—C1	120.7 (3)	N1—C9—H9	119.8
C3—C2—H2A	119.7	C8—C9—H9	119.8
C1—C2—H2A	119.7	N3—C10—N2	115.8 (2)
C2—C3—C4	119.2 (3)	N3—C10—S1	124.5 (2)
C2—C3—H3A	120.4	N2—C10—S1	119.7 (2)
C4—C3—H3A	120.4	N3—C11—H11A	109.5
C5—C4—C3	121.0 (3)	N3—C11—H11B	109.5
C5—C4—H4	119.5	H11A—C11—H11B	109.5
C3—C4—H4	119.5	N3—C11—H11C	109.5
C4—C5—C6	120.9 (3)	H11A—C11—H11C	109.5
C4—C5—H5	119.6	H11B—C11—H11C	109.5
C6—C5—H5	119.6		
			
C9—N1—N2—C10	178.3 (2)	C1—C6—C7—C8	178.7 (2)
C6—C1—C2—C3	−0.6 (4)	C5—C6—C7—C8	−1.9 (4)
C1—C2—C3—C4	−0.2 (4)	C6—C7—C8—C9	178.0 (2)
C2—C3—C4—C5	0.3 (5)	N2—N1—C9—C8	178.6 (2)
C3—C4—C5—C6	0.5 (5)	C7—C8—C9—N1	179.7 (2)
C2—C1—C6—C5	1.4 (4)	C11—N3—C10—N2	179.2 (3)
C2—C1—C6—C7	−179.2 (2)	C11—N3—C10—S1	−2.5 (4)
C4—C5—C6—C1	−1.3 (4)	N1—N2—C10—N3	−4.7 (4)
C4—C5—C6—C7	179.3 (3)	N1—N2—C10—S1	176.89 (18)

### Hydrogen-bond geometry (Å, º)

**Table:**

*D*—H···*A*	*D*—H	H···*A*	*D*···*A*	*D*—H···*A*
N2—H2···S1^i^	0.86	2.67	3.368 (3)	139
N3—H3···N1	0.86	2.20	2.604 (6)	109
C11—H11*C*···S1	0.96	2.75	3.108 (6)	103

Symmetry code: (i) −*x*, −*y*, −*z*+2.
